# Mechanistic correlation between water infiltration and framework hydrophilicity in MFI zeolites

**DOI:** 10.1038/s41598-019-54751-5

**Published:** 2019-12-05

**Authors:** Matteo Fasano, Alessio Bevilacqua, Eliodoro Chiavazzo, Thomas Humplik, Pietro Asinari

**Affiliations:** 1 Department of Energy, Politecnico di Torino, Corso Duca degli Abruzzi 24, Torino, 10129 Italy; 2 Department of Mechanical Engineering, Massachusetts Institute of Technology, Cambridge, MA USA

**Keywords:** Mechanical engineering, Atomistic models

## Abstract

Hydrophobic zeolites are nanoporous materials that are attracting an increasing interest, especially for catalysis, desalination, energy storage and biomedical applications. Nevertheless, a more profound understanding and control of water infiltration in their nanopores is still desirable to rationally design zeolite-based materials with tailored properties. In this work, both atomistic simulations and previous experimental data are employed to investigate water infiltration in hydrophobic MFI zeolites with different concentration of hydrophilic defects. Results show that limited concentrations of defects (e.g. 1%) induce a change in the shape of infiltration isotherms (from type-V to type-I), which denotes a sharp passage from typical hydrophobic to hydrophilic behavior. A correlation parametrized on both energy and geometric characteristics of the zeolite (infiltration model) is then adopted to interpolate the infiltration isotherms data by means of a limited number of physically-meaningful parameters. Finally, the infiltration model is combined with the water-zeolite interaction energy computed by simulations to correlate the water intrusion mechanism with the atomistic details of the zeolite crystal, such as defects concentration, distribution and hydrophilicity. The suggested methodology may allow a faster (more than one order of magnitude) and more systematic preliminary computational screening of innovative zeolite-based materials for energy storage, desalination and biomedical purposes.

## Introduction

The peculiar properties of water confined by hydrophobic surfaces at the nanoscale are important in several areas of science and technology^1–7^. While bulk water molecules experience strong mutual attraction due to hydrogen bonding, the lack of hydrogen bonds between water and hydrophobic surfaces causes a decrease in the overall interaction energy^8^. Such effect is particularly pronounced in nanoporous hydrophobic materials (e.g. carbon nanotubes, zeolites such silicalite-I, chabazite or ZSM-5)^9^, where the liquid is in contact with an ultra large and highly developed surface^10^. Hydrophobicity has been extensively studied, but a complete comprehension of many aspects is still elusive^4^. For example, despite numerous experiments of water occupancy in nonpolar nanopores^11,12^, a complete understanding of the thermodynamics of water in nonpolar cavities at different temperatures has not yet been achieved^8^.

In case of hydrophobic framework, pore filling typically takes place at pressures higher than the saturated vapor pressure (*p* > *p*_0_, namely infiltration process), being the intruded water in the liquid phase^13^. Under these conditions, the quantity of intruded water and the pressure at which infiltration starts to occur (infiltration pressure) closely depend on the pore geometry and diameter, as well as on the presence of polar sites^1^. The intrusion of water in hydrophobic nanopores can be either reversible or irreversible, where the irreversibility may arise from the creation of defects within the nanoporous structure^14^. Moreover, the infiltration process can be exothermic (e.g. water in faujasite) or endothermic (e.g. water in silicalite-I), according to the conformation of the pore network^15^.

The distinctive properties of water molecules interacting with hydrophobic surfaces are relevant in a broad range of biomedical and engineering applications. For example, nanomedicine widely exploits the mass transport properties of nanoconfined water to design imaging and/or therapeutic nanoconstructs^16–18^, and confined water is a key factor in the functioning of biological channels and proteins^19–21^. In the engineering field, the interaction between water and hydrophobic nanopores underpins the development of separation, catalysis, nanofabrication, energy conversion/storage and purification processes^22–26^. Within the large group of hydrophobic nanoporous materials, zeolites are attracting an increasing interest, since geometry, adsorption characteristics, catalytic behavior and ion exchange capability can be tailored to a specific application by tuning the chemical composition, framework structure and concentration of defects^27^. In particular, the sub-nanometer pore size of hydrophobic zeolites has been proposed as a medium for energy storage and water desalination.

First, systems consisting of water and hydrophobic zeolites have the potential to store and then release mechanical energy, as well as to transform or dissipate it^5,28^. Hydrophobic zeolites can be infiltrated by liquid water when the applied hydrostatic pressure is higher than the capillary one, as computed from the Laplace-Washburn relation^5^. The applied pressure can be therefore regulated to infiltrate/expel liquid water from the nanopores, with a consequent transformation of mechanical into interfacial energy (and vice versa). While a reversible transformation allows to implement molecular springs and actuators, irreversible ones lead to dampers and shock absorbers^1,13,28,29^. Zeolite-based materials are thus considered as possible constituents of energy storage/dissipation systems with performance 1-2 orders of magnitude higher than traditional materials^30^; hence, many experimental^5,31,32^ and modeling^8,33,34^ studies have been devoted to relate water infiltration process with the physical-chemical features of the system.

Second, hydrophobic zeolites have been studied as elements of innovative membranes for Reverse Osmosis (RO) desalination. In fact, despite RO technologies have been widely commercialized, innovative materials for RO membranes are required to increase both fouling resistance and energy efficiency of the desalination process^35^. Mordenite Framework Inverted (MFI) zeolites have nanopores with diameters such that only water molecules can permeate through, while hydrated salt ions are totally rejected. Therefore, MFI zeolites have been studied as promising materials for RO membranes^27,36^; however, a commercial widespread of zeolites is still slowed down by a limited comprehension of how nanoscale characteristics of the network of pores (e.g. topology, concentration of defects, hydrophilicity) and the resulting water transport properties of the membranes are correlated^27,36,37^. Atomistic simulations may support a more systematic and fundamental analysis of water intrusion in zeolites with tunable hydrophobicity/hydrophilicity, with the possibility to provide design guidelines for zeolite-based energy storage/dissipation or RO devices^38,39^.

Regarding the numerical simulation of water intrusion in MFI zeolites, several previous works focused on the Monte Carlo simulation of water adsorption on pristine^40^ (fully hydrophobic) or defected^41,42^ (partially hydrophilic) MFI crystals, therefore not studying the water infiltration process that occurs at pressures larger than the saturation one. Other articles presented the water diffusion in pristine MFI at different pressures, without any insight on the infiltration process^43–45^. Some studies on water infiltration in MFI, instead, did not computed the related infiltration isotherms: for instance, Liu *et al*.^46^ and Rassoulinejad-Mousavi *et al*.^47^ investigated only the permeability and salt rejection capability of pristine MFI membranes. Finally, some articles measured the infiltration isotherms of water in MFI zeolites by atomistic simulations, but without providing any mechanistic understanding on how they are affected by the surface characteristics of the nanopores. This is the case of the works by Desbiens *et al*.^34^ and Santoro *et al*.^48^ (infiltration isotherms of water in pristine MFI by Monte Carlo simulations), Trzpit *et al*.^33^ and Cailliez *et al*.^8^ (infiltration isotherms of water in MFI zeolites with different concentrations of defects – thus hydrophilicity – by Monte Carlo simulations), and Vaarstra *et al*.^49^ (infiltration isotherms of water in MFI zeolites with different hydrophilicity by molecular dynamics simulations).

In this work, the mechanism of water infiltration in crystals of MFI zeolites is investigated by atomistic simulations and validated by previous experimental data. The infiltration isotherms of water in MFI crystals are computed by a numerical protocol available from the literature^38^; whereas, the water-zeolite interactions are estimated via a new simulation setup based on periodic zeolite crystals. The water-zeolite interaction energy is here regulated by different concentrations of hydrophilic defects, which are progressively introduced in the initially hydrophobic MFI framework. Both experimental evidence from literature and Molecular Dynamics (MD) simulations demonstrate that a correlation parametrized on both energy and geometric characteristics of the zeolite can interpolate the infiltration isotherms data of water in MFI zeolites with a minimum number of physically-meaningful parameters. The resulting new model-driven approach for the exploration of novel nanoporous materials with tunable infiltration properties is of general applicability in several applications, spanning from engineering to biomedical fields. As an example, the methodology is benchmarked by two relevant test-cases, namely zeolite samples with either strongly hydrophilic or locally concentrated crystal defects.

## Results

### Fitting infiltration isotherms from experiments

**Figure 1:**
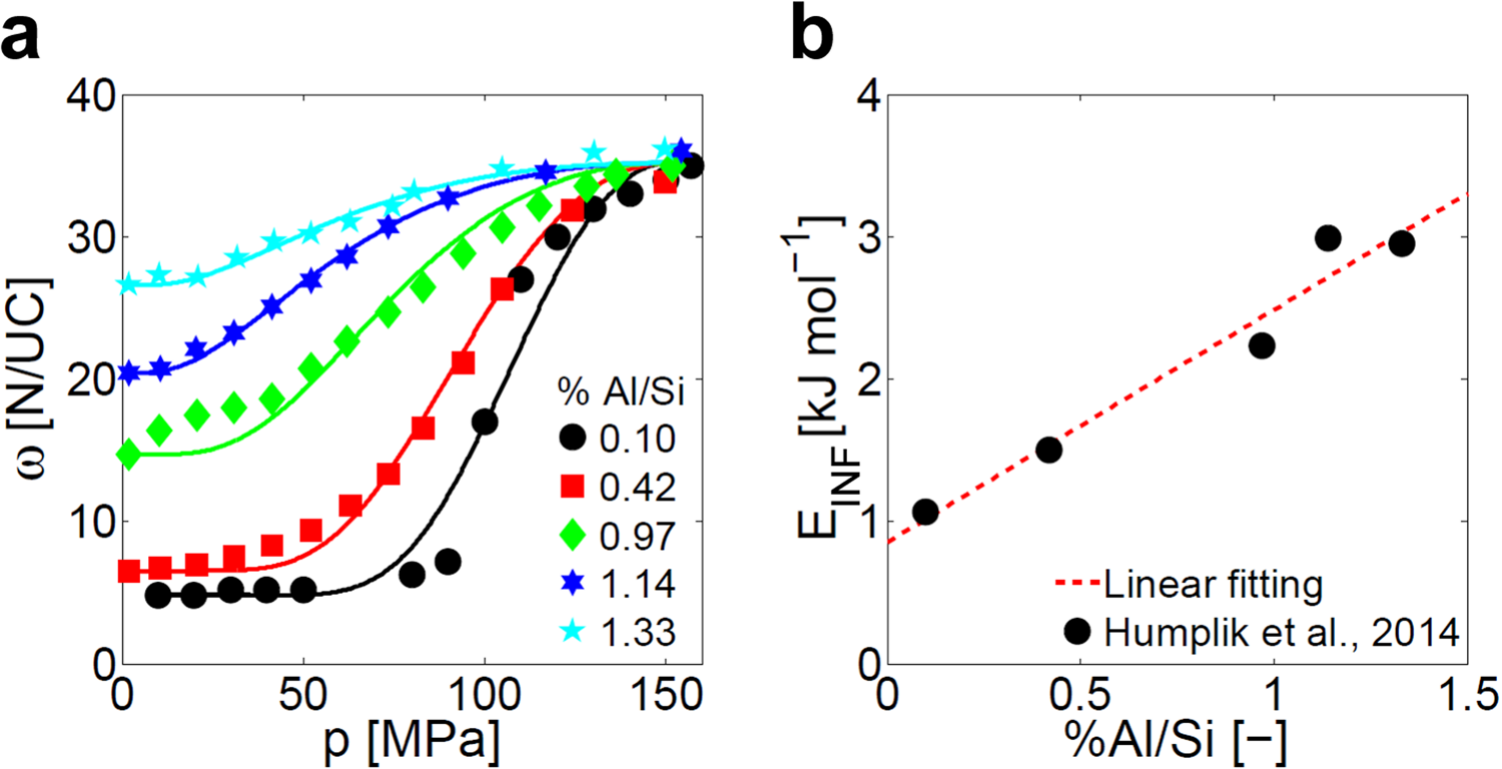
Experimental infiltration isotherms of water in defected MFI zeolites. Intruded water molecules (N) per unit cell (UC) of MFI zeolite (*ω*, expressed in N/UC units) at different pressures (*p*): experimental infiltration isotherms (taken from ref. ^27^) and infiltration model fitting (Eq. 1) are shown. **(a)** Effect of the concentration of hydrophilic defects (percent substitution of Si by Al atoms in the crystal framework) on the infiltration isotherms of water in MFI zeolites: experiments (symbols) and model fittings (solid lines) are reported. **(b)** Optimal values of *E*_INF_ (infiltration energy parameter, Eq. 1) for the MFI zeolites with different concentration of defects reported in **a**.

It has been experimentally observed that liquid water interacts with the hydrophobic structure of pristine MFI zeolite (also known as silicalite-I) with a three-step process (see black dots in Fig. 1a)^27,50^. First, water cannot intrude the nanoporous framework at pressures lower than the infiltration one, which is typically around 90 MPa. Second, water molecules infiltrate into the silicalite-I structure within a limited range of pressures (between 90 and 110 MPa), giving rise to an endothermic effect. Third, over 110 MPa, water molecules are further compressed in the zeolite pores until the maximum framework capacity of the host structure is eventually achieved, as a consequence of steric hindrance between the molecules^1^.

Humplik and colleagues^27^ experimentally characterized MFI zeolites with different concentration of hydrophilic defects, by substituting Si atoms with Al ones and thus creating silanol nests within the pristine MFI structure. On the one side, the internal structure of the pores was unaffected by the introduction of defects, as demonstrated by the similar XRD patterns for all the silicalite-I and defected MFI samples (see the Fig. 1 in ref. ^27^). On the other side, small concentrations of hydrophilic defects led to lower infiltration pressures and dramatically different shapes of infiltration isotherms, due to the alteration of the surface chemistry of the pores. Those previous experimental results are recalled in Fig. 1a (colored symbols): the infiltration isotherms of water in MFI zeolites with up to 0.5% Al/Si substitutions show a type-V shape, namely the typical behavior of hydrophobic frameworks. Under such conditions, water-water interactions are higher than water-zeolite ones and, therefore, water condensation in the MFI pores takes place by the collapse of homogeneously nucleated clusters of infiltrated water molecules^8,34,51^. A progressive transformation from type-V to type-I infiltration isotherms is observed with Al/Si substitutions larger than 0.5%. In these cases, water-zeolite interactions are dominant in the process of condensation, therefore inducing a heterogeneous (and more gradual) nucleation of solvent molecules close to the hydrophilic (*i.e*., defected) regions of the crystal^8,52–54^. In other words, pore filling in hydrophilic MFI zeolites starts with the water vapor adsorption at pressures eventually lower than *p*_0_, similarly to what observed in the nanoporous materials for sorption heat storage^55–57^. With increasing hydrophobic behavior, instead, the pore filling pressure increases, and it becomes orders of magnitude higher than the saturated vapor pressure^13^; under these conditions, the intruded water is liquid and pore filling occurs as an infiltration process^58^.

The Dubinin-Astakhov model (D-A) is a correlation that has been demonstrated to underpin a broad variety of adsorption processes^59–61^. Here, an empirical correlation similar to the D-A model is introduced for interpolating the infiltration isotherms data with a minimal number of parameters related to the characteristics of the nanoporous material, namely:

1 $$\frac{\omega -{\omega }_{{\rm{m}}}}{{\omega }_{{\rm{M}}}-{\omega }_{{\rm{m}}}}=\exp [-{(-\frac{{k}_{{\rm{B}}}{N}_{{\rm{A}}}T}{{E}_{{\rm{INF}}}}\mathrm{ln}\frac{p}{{p}_{{\rm{M}}}})}^{{n}_{{\rm{INF}}}}\,],$$

where *ω* is the number of intruded water molecules per unit cell of nanoporous material (N/UC), *p* is the water pressure and *T* is the system temperature (*k*_B_ = 1.38 × 10^−23^ J K^−1^; *N*_A_ = 6.022 × 10^23^ mol^−1^). Analogously to the D-A model, the parameters *E*_INF_ and *n*_INF_ should depend on the sorbate-sorbent (water-zeolite, in this case) interaction energy and the crystal structure of the sorbent, respectively. Furthermore, the infiltration model in Eq. 1 includes also the maximum framework capacities of the adsorption (*ω*_m_ = *ω*(*p*_0_)) and infiltration (*ω*_M_ = *ω*(*p*_M_)) phase, being *p*_M_ the water pressure at which *ω*_M_ is eventually achieved. While *ω*_m_, *ω*_M_ and *p*_M_ are quantities that can be easily extrapolated from direct measures, *E*_INF_ and *n*_INF_ should be obtained by fitting Eq. 1 to *ω* − *p* isotherms.

The XRD patterns reported in the previous work by Humplik and colleagues^27^ confirm that the crystal structure of the zeolite samples in Fig. 1a can be considered as invariant in the considered range of concentrations of defects, at least as a first approximation. Therefore, it is possible to consider *n*_INF_ as a quantity independent from the concentration of defects, being *E*_INF_ the sole parameter affected by the increasing framework hydrophilicity. The optimization of *E*_INF_ and *n*_INF_ to fit the experimental results with Eq. 1 is then performed, and the best-fitting curves are reported in Fig. 1a as solid lines (*R*^2^ > 0.95). Results show that *n*_INF_ = 2 is the optimal model exponent for the considered zeolites, while the best-fitted *E*_INF_ values (black dots in Fig. 1b) clearly highlight their dependence on framework hydrophilicity. In particular, the relation between *E*_INF_ and the concentration of defects can be accurately fitted by a linear function (*R*^2^ = 0.94, red dashed line in Fig. 1b), namely *E*_INF_ = *a*_1_⋅%*Al*/*Si* + *a*_2_ with *a*_1_ = 1632 J mol^−1^ and *a*_2_ = 857 J mol^−1^. Hence, *E*_INF_ appears as a multiscale parameter that links the fundamental mechanism of water intrusion in the zeolite pores (*i.e*., water-zeolite nonbonded interactions) with the macroscopic, effective properties of the zeolite sample (*i.e*., infiltration isotherms).

### Zeolite membrane simulations

**Figure 2:**
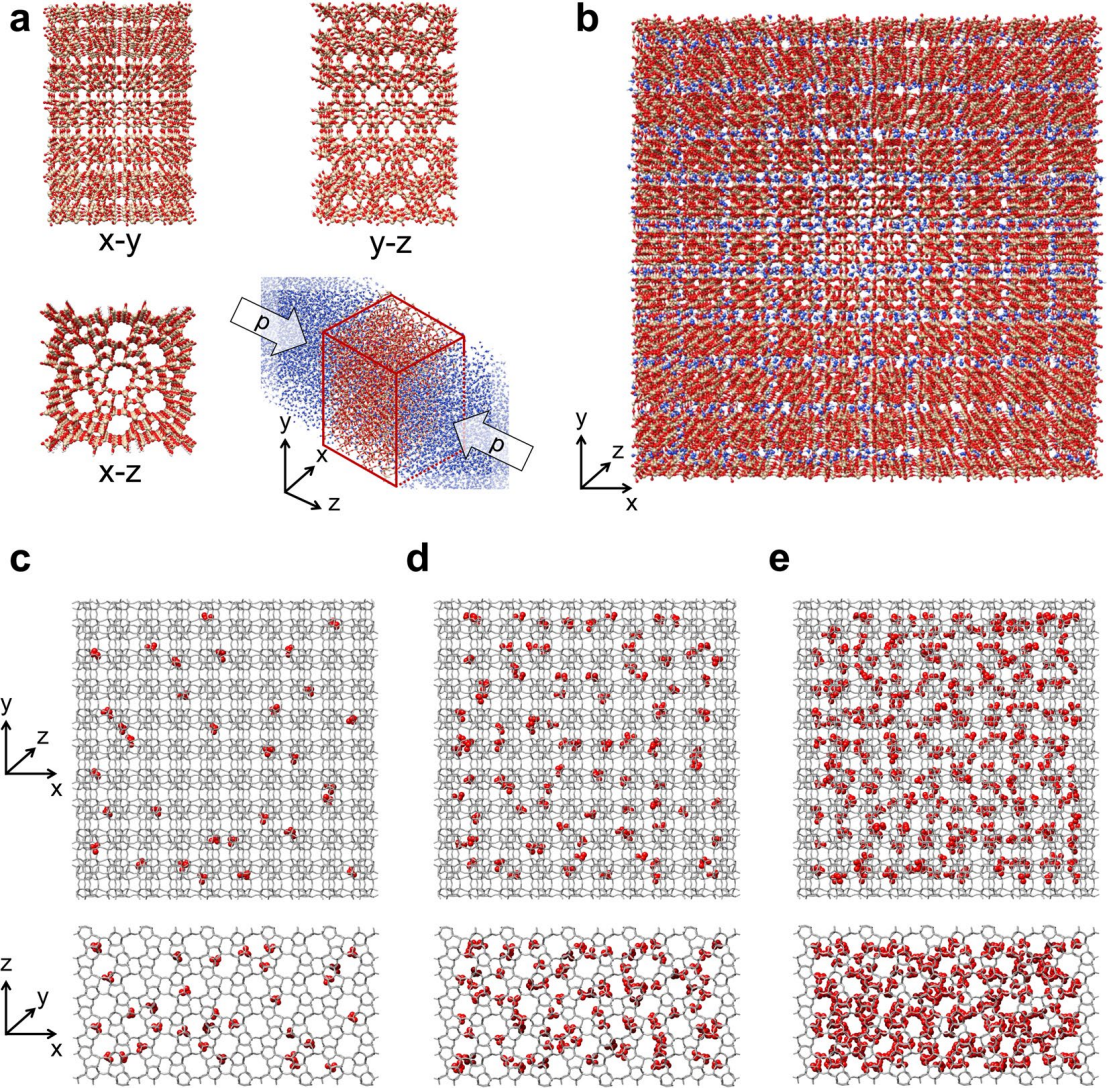
Schematics of the simulation domains. The MFI zeolite crystal (red/tan) and water molecules (blue) analyzed by Molecular Dynamics (MD) simulations are represented. **(a)** Infiltration experiments by MD simulations: MFI zeolite membrane is pictured before (*x* − *y*, *y* − *z* and *x* − *z* views) and after the water infiltration (axonometric view) induced by a simulated solvent pressure (*p*). **(b)** Computation of the water-zeolite interaction energies in a bulk zeolite crystal periodic along *x*, *y* and *z* directions. (**c**) Random distribution of defects in the simulated bulk zeolite crystals with 0.33%, (**d**) 0.89% and (**e**) 3.06% equivalent Al/Si substitution. Hydrogen atoms belonging to silanol nests are magnified and highlighted in red, while all the other atoms are gray. Frontal (*x* − *y*) and top (*x* − *z*) views are both reported for each configuration. Rendering pictures are made with UCSF Chimera^73^.

Molecular dynamics simulations are then carried out to reproduce the infiltration behavior of the defected MFI zeolites observed by experiments and, consequently, to investigate the water intrusion at the atomistic scale. To this purpose, a 4 × 6 × 34 nm^3^ computational domain is first simulated, where an MFI membrane with 4 × 6 × 4 nm^3^ dimensions is placed in the middle of a water box (Fig. 2a). The adopted force field is made of bonded and nonbonded (Lennard-Jones and Coulomb) interactions, and it has been optimized in previous works^38,39^. The pores of the membrane are initially empty; increasing pressures are then applied along *z* axis to induce water infiltration and thus reproduce the characteristic infiltration isotherm. The hydrophobicity of the pristine MFI framework is progressively decreased by introducing silanol defects. The silanols insertion in the MFI crystal qualitatively mimics the hydrophilicity enhancement obtained in the experiments by substituting silicon atoms with aluminum ones^8,33,38,39^. Partial charges of silanol nests are set to *q*_*H*_ = 0.45 e and *q*_*O*_ = −0.9 e, where e is the elementary charge^8,38^.

**Figure 3:**
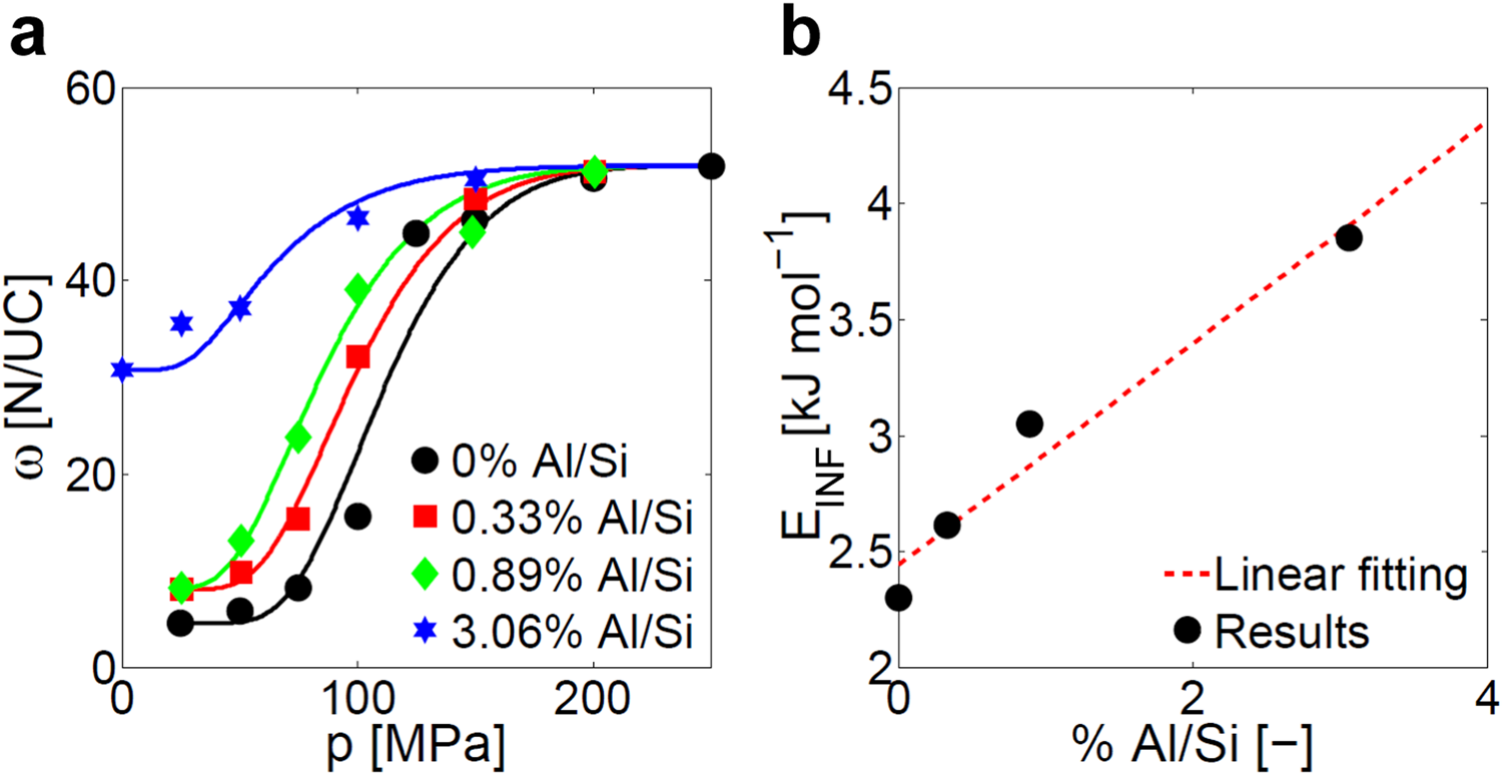
Simulated infiltration isotherms of water in defected MFI zeolites. (**a**) Infiltration isotherms of water in MFI zeolites with different concentration of defects (from 0 to 3.06% Al/Si): MD results (symbols) and optimized infiltration models (solid lines, Eq. 1) are both represented. **(b)** Optimized values of the infiltration energy parameter (*E*_INF_, Eq. 1) for the MFI zeolites with different concentration of defects in **a**, as obtained from the best-fitting of MD simulations.

Starting from the preliminary results reported in our previous work^38^, MFI membranes are simulated with random distributions of various concentrations of defects, which are equivalent to 0%, 0.33%, 0.89% and 3.06% substitutions of Si atoms by Al ones (%Al/Si). Coherently with experiments from the literature^35^, simulations (symbols in Fig. 3a) show that more hydrophilic membranes are characterized by lower infiltration pressures and type-I infiltration isotherms.

As previously assumed, the crystal structure of the MFI membranes can be considered as invariant in the simulated configurations. Again, Eq. 1 can be fitted to the numerical infiltration isotherms by considering that *n*_INF_ is constant for the tested configurations, while *E*_INF_ depends on the concentration of defects. A genetic algorithm is employed to optimize the model fitting on the results from infiltration simulations, and the best-fitted curves (*R*^2^ > 0.90) are reported in Fig. 3a. The fitting procedure finds *n*_INF_ = 3.14 as the most accurate parameter for the simulated zeolite membranes; the optimized values of *E*_INF_, instead, are depicted in Fig. 3b (black dots) for different defects concentration. On the one hand, *n*_INF_ is larger than the value found by fitting experimental infiltration isotherms. This evidence highlights the non-ideality of the experimental structure, which may lead to discrepancies between numerical and experimental results. In fact, the experimental analysis of zeolite samples presenting pore blockage/narrowing, surface barriers or crystal contaminations may alter the accessible pore volume and thus the *n*_INF_ value^39^, as also proved by the different maximum framework capacity (*ω*_M_) obtained in the simulations (52 N/UC) with respect to the Humplik’s experiments (35 N/UC)^38,50^. On the other hand, *E*_INF_ is again observed to be proportional to the concentration of defects, because of the enhanced water-zeolite interactions provided by the more hydrophilic surface of nanopores. In Fig. 3b, the *E*_INF_ values obtained from the MD simulations are accurately fitted (*R*^2^ = 0.94) by a linear function (red dashed line, *E*_INF_ = *a*_1_⋅%*Al*/*Si* + *a*_2_, with *a*_1_ = 478 J mol^−1^ and *a*_2_ = 2443 J mol^−1^).

Hence, simulation and previous experimental evidence demonstrate that Eq. 1 can accurately fit the infiltration isotherms of water in MFI zeolites at varying hydrophilicity. In particular, while *n*_INF_ only depends on the geometrical characteristics of the network of nanopores, *E*_INF_ scales with the magnitude of the interaction potential between water and nanopores, namely zeolite hydrophilicity. Therefore, in principle, the characteristic infiltration isotherms of zeolite membranes should be predictable *a priori* from the fluid-crystal nonbonded interactions.

### Bulk zeolite simulations

To better investigate the mechanistic relation between water-zeolite interaction potentials and *E*_INF_ (energy parameter in Eq. 1), the average nonbonded interaction energies per infiltrated water molecule are computed for MFI crystals at different pore hydration (*ϑ*_M_ = *ω*/*ω*_M_, being *ω*_M_ = 52 N/UC in the simulated cases) and concentration of defects.

To this purpose, a simulation domain containing a 10.0 × 9.9 × 5.4 nm^3^ zeolite crystal is built from the unit cell of silicalite-I, with periodic boundary conditions applied along the three Cartesian axis (see Fig. 2b). Again, hydrophilic zeolites are obtained by introducing silanol nests in the pristine MFI framework, following a random distribution among the possible crystallographic sites (see Fig. 2c–e). The dry crystal is first energy minimized; then, water molecules are introduced into the zeolite pores by means of a Monte Carlo-like algorithm. The considered number of water molecules is chosen to span the whole interval of pressures studied in the infiltration experiments (Fig. 3a), that is *ω* = 5, 10, 30, 50 water molecules per unit cell. The mean interaction energies arising from both Coulomb and Lennard-Jones potentials are computed at equilibrium conditions.

**Figure 4:**
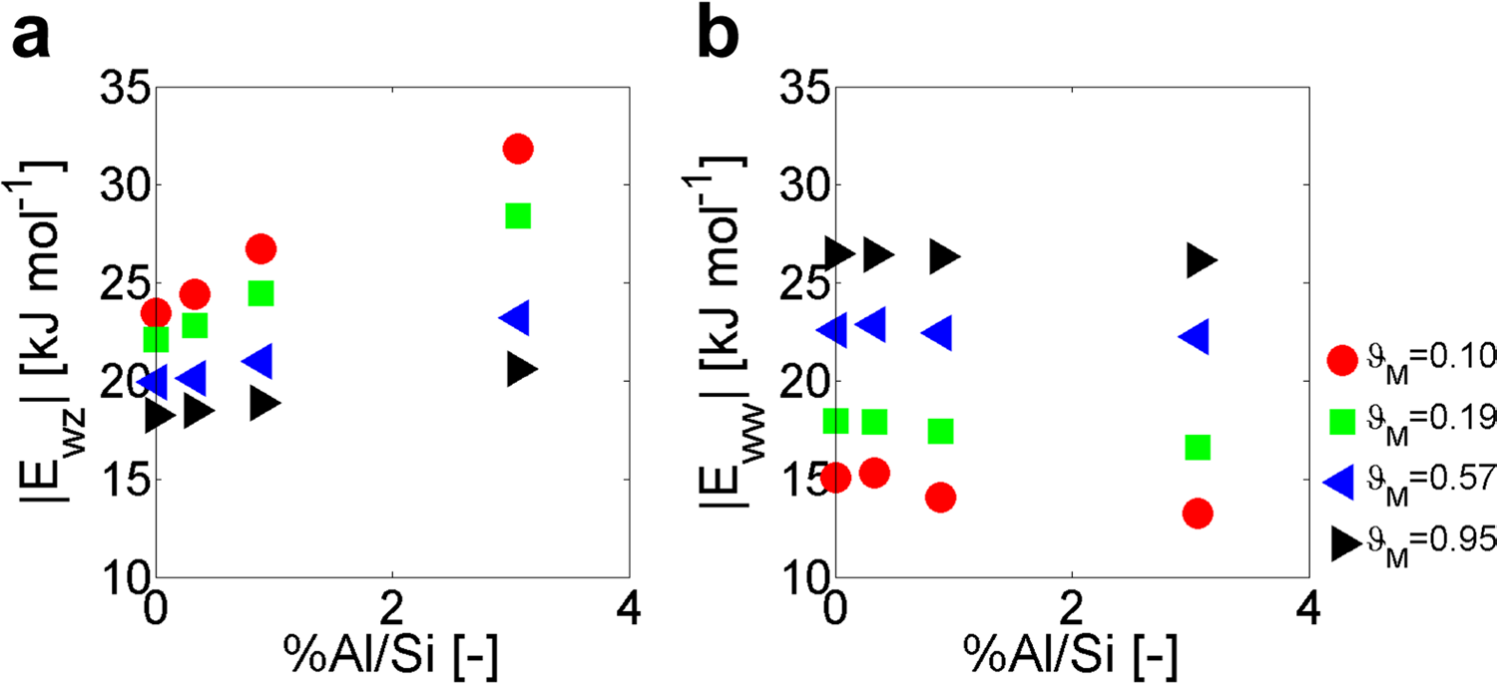
Specific interaction energies from atomistic simulations. Specific interaction energies vs. concentration of defects at different zeolite hydrations (*ϑ*_M_). **(a)** Water-zeolite (*E*_wz_) specific interaction energy (see Eq. 2). **(b)** Water-water (*E*_ww_) specific interaction energy (see Eq. 3).

On the one side, the water-zeolite specific interaction energy can be defined as:

2 $${E}_{{\rm{wz}}}=\frac{{U}_{{\rm{LJ}}-{\rm{wz}}}+{U}_{{\rm{C}}-{\rm{wz}}}}{\omega {N}_{{\rm{UC}}}},$$

where *N*_UC_ is the number of unit cells in the crystal (100 in the simulated cases); *U*_LJ−wz_ and *U*_C−wz_ are the overall water-zeolite interaction energies averaged along the simulated trajectory due to Lennard-Jones and Coulomb potentials, respectively^39^. Note that the interaction energies are computed only for water molecules completely intruded in the zeolite. *E*_wz_ represents the effective nonbonded potential exerted by the surface of zeolite nanopores on each infiltrated water molecule, on average. *E*_wz_ shows negative values due to the attractive nature of water-zeolite interactions within the MFI framework; however, for clarity, *E*_wz_ is reported in absolute terms in the following analyses. In Fig. 4a, a linear dependence between *E*_wz_ and the concentration of defects can be noticed, therefore denoting a clear correlation between *E*_wz_ and zeolite hydrophilicity. Figure 4a also shows decreasing slopes for the *E*_wz_ − %Al/Si linear relations with larger pore hydration, because of the dominating effect of water-water interactions at high pore hydration. In fact, the increase in nanopore hydration implies that a smaller fraction of the overall volume of intruded water is in contact with the nanopore surface, therefore progressively lowering the *E*_wz_ value.

On the other side, the water-water specific interaction energy can be analogously defined as:

3 $${E}_{{\rm{ww}}}=\frac{{U}_{{\rm{LJ}}-{\rm{ww}}}+{U}_{{\rm{C}}-{\rm{ww}}}}{\omega {N}_{{\rm{UC}}}},$$

being *U*_LJ−ww_ and *U*_C−ww_ the overall water-water interaction energies averaged along the simulated trajectory due to Lennard-Jones and Coulomb potentials, respectively^39^. Again, *E*_ww_ shows negative values thus attractive interactions, but it is reported in absolute terms in the followings. Results in Fig. 4b show that the absolute water-water interaction energy tends to increase with pore hydration, mainly because of the higher number of H-bonds between intruded water molecules. In contrast to *E*_wz_, *E*_ww_ appears to be almost insensible to pore hydrophilicity, especially at large hydration regimes (*ϑ*_*M*_ → 1).

### Mechanistic infiltration isotherms

**Figure 5:**
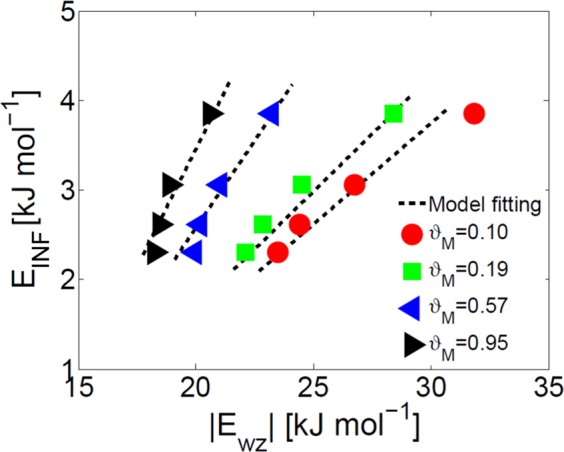
Correlation between specific interaction energy and infiltration energy parameter. Correlation between infiltration energy parameter (*E*_INF_, Eq. 1), water-zeolite specific interaction energy (*E*_wz_, Eq. 2) and pore hydration (*ϑ*_M_) of the defected MFI crystals simulated by MD. Results from simulations at different pore hydration (symbols) are accurately fitted (*R*^2^ > 0.91) by the model presented in Eq. 4 (dotted lines).

The drastically different water infiltration mechanism experimentally and numerically observed in zeolites with different framework hydrophilicity can be ascribed to the water-zeolite interactions and, therefore, *E*_wz_ appears as the most suitable link between the atomistic details and overall properties of zeolite crystals. Coherently, Fig. 5 shows a clear correlation between *E*_INF_ and *E*_wz_, being *E*_INF_ directly proportional to the water-zeolite interaction energy dictated by the concentration of defects. Note that the slope of the correlation between *E*_INF_ and *E*_wz_ scales with *ϑ*_*M*_. In fact, the *E*_wz_/*E*_ww_ ratio is inversely proportional to pore hydration (see Fig. 4) and, therefore, limited absolute increments of *E*_wz_ at *ϑ*_*M*_ → 1 lead to sharper *E*_INF_ increases. Hence, an accurate description of the nanoscale properties of zeolite (*E*_wz_) is in principle enough to predict its macroscopic properties (*E*_INF_, namely infiltration isotherm of water in zeolite) by means of a multiscale correlation. For example, let us introduce a semi-empirical correlation between *E*_INF_, *E*_wz_ and *ϑ*_M_, namely

4 $${E}_{{\rm{INF}}}=(k{\vartheta }_{{\rm{M}}}+\alpha )(|{E}_{{\rm{wz}}}|-|{E}_{{\rm{wz}}}^{0}|),$$

being *k*, *α* and *E*_wz_^0^ rescaling coefficients. In Fig. 5, the dashed black lines indicate that the *E*_INF_ parameters found by MD for several MFI crystals with growing concentration of hydrophilic defects can be accurately fitted (*R*^2^ > 0.91) by Eq. 4, with optimized coefficients *k* = 0.35, *α* = 0.19 and |*E*_wz_^0^| = 13.42 kJ mol^−1^. Hence, at least for the considered MD model, Eq. 4 allows predicting the characteristic *E*_INF_ for a defected MFI crystal by only evaluating the average water-zeolite interaction energy at a certain pore hydration; *E*_INF_ and the infiltration model in Eq. 1 could be then used to estimate the whole infiltration isotherms of the MFI zeolite.

**Figure 6:**
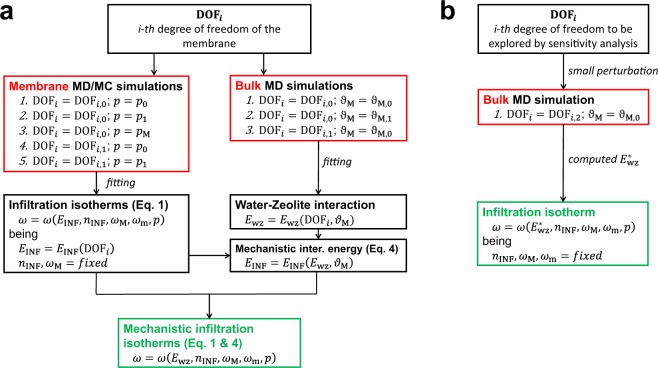
Schematics of the suggested methodology. (**a)** Tuning the correlation between infiltration isotherms of water in zeolite and water-zeolite specific interaction energies. Note that DOF_*i*_ is the *i-th* degree of freedom of the zeolite crystal (e.g., concentration of defects, distribution, type, etc.) to be explored; whereas, DOF_*i*,0_ and DOF_*i*,1_ are different values of DOF_*i*_. Similarly, *p*_0_ and *p*_1_ are different solvent pressures; whereas, *ϑ*_M,0_ and *ϑ*_M,1_ pores hydrations. **(b)** Sensitivity analysis that can be performed by exploiting the correlation between the infiltration isotherms and specific interaction energies, at least in the limit of small perturbations of the original configuration (*i.e*., *n*_INF_, *ω*_M_, *ω*_m_ approximately constant). In the schematics, simulation steps are contained in red boxes, while final outputs in green ones.

Summarizing, a comprehensive methodology to investigate the effect of the possible degrees of freedom (DOF, e.g. defects concentration and type, pore occlusions, etc.) of zeolite-based membranes on their water infiltration behavior can be then outlined. Here, the more general aim is providing a systematic approach for a fast computational exploration of novel nanoporous materials, with immediate applications in energy or desalination fields. The methodology can be subdivided into two distinct phases, namely (i) tuning the multiscale correlations and (ii) performing the sensitivity analyses. Figure 6a schematically depicts the first phase. In detail, a minimum set of 5 molecular dynamics or Monte Carlo simulations of the considered membrane (see the configuration in Fig. 2a) and 3 molecular dynamics runs of the corresponding bulk nanoporous crystal (see the configuration in Fig. 2b) are needed to tune the correlations allowing a systematic DOF exploration, namely *ω* = *ω*(*E*_INF_, *n*_INF_, *ω*_M_, *ω*_m_, *p*) (Eq. 1) and *E*_INF_ = *E*_INF_(*E*_wz_, *ϑ*_*M*_) (Eq. 4**)**. The mechanistic correlation between the atomistic details of the MFI crystal and the corresponding infiltration isotherms can be subsequently determined, namely *ω* = *ω*(*E*_wz_, *n*_INF_, *ω*_M_, *ω*_m_, *p*). Second, Fig. 6b shows how sensitivity analyses can be then easily performed by means of a limited amount of molecular dynamics simulations, at least in the limit of small perturbations of the original setup (*i.e*., *n*_INF_, *ω*_M_, *ω*_m_ approximately constant). Note that this hypothesis requires that the geometrical characteristics of pores (that is, zeolite framework) are not significantly altered by DOF variation. Infiltration isotherms can be finally estimated by $\omega =\omega ({E}_{{\rm{wz}}}^{\ast },{n}_{{\rm{INF}}},{\omega }_{{\rm{M}}},{\omega }_{{\rm{m}}},p)$, where ${E}_{{\rm{wz}}}^{\ast }$ is measured by a sole MD simulation of the bulk zeolite crystal with the DOF value to be tested (DOF_*i*_ = DOF_*i*,2_). Clearly, the procedure in Fig. 6b allows a drastic reduction in the computational burden otherwise required for the complete procedure, which is instead depicted in Fig. 6a.

## Discussion

The methodology outlined in Fig. 6 allows estimating the characteristic infiltration isotherms of water in zeolite crystals by only resorting on a few simulations. This would indeed reduce the computational burden for exploring and optimizing the degrees of freedom of the zeolite, therefore paving the way to a model-driven design of novel materials for RO or energy applications. Let us consider two exemplificative cases to test the prediction capability of the methodology reported in Fig. 6b. In these examples, the distribution and hydrophilicity of the defects in one of the MFI zeolites studied so far (0.89% Al/Si) are modified, to assess their effect on infiltration isotherms.

**Figure 7:**
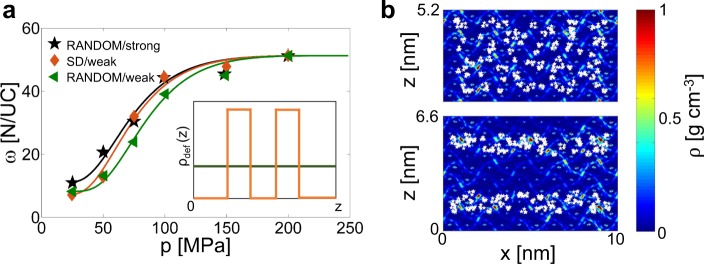
Sensitivity analyses on defects characteristics. (**a)** Infiltration isotherms of water in a crystal made of defected MFI (0.89% Al/Si): green triangles and line stand for the base case (random distribution of defects; “weak” partial charges for silanols); black stars and line for the case with more hydrophilic defects (random distribution of defects; “strong” partial charges for silanols); orange rhombus and line for the case with SD defect distribution (stripe distribution of defects; “weak” partial charges of silanols). MD results (symbols) and optimized infiltration models (solid lines, *R*^2^ > 0.85) are both shown. In the inset, the random (green line) and SD (orange line) 1-dimensional distribution of defects are schematically depicted. **(b)** 2D (*y*-axis averaged) density distributions of water within MFI crystals (0.89% Al/Si, *ω* = 10 N/UC) with different defects arrangements, namely random (upper panel) or SD (lower panel). White stars represent the position of defects in the zeolite crystal, whereas the time-averaged water density is colored from blue (lower densities) to red (higher densities).

In the first case, the partial charge of silanol defects are changed from *q*_H_ = 0.45 e and *q*_O_ = −0.9 e (“weak” configuration) to *q*_H_ = 0.65 e and *q*_O_ = −1.1 e (“strong” configuration). According to Fig. 6b, a sole MD simulation of the zeolite bulk crystal (*ϑ*_M_ = 0.95, near maximum pore hydration) is needed to compute *E*_wz_, which here takes the value of *E*_wz_ = −20.24 kJ mol^−1^. This value can be then used to estimate *E*_INF_ and thus the complete infiltration isotherm by Eq. 4: *E*_INF_ = 3.56 kJ mol^−1^, which is 17% higher compared to the “weak” case (3.05 kJ mol^−1^). For validating the predicted infiltration isotherm of the MFI crystal with “strong” defects, a complete set of MD infiltration experiments is then performed over the 25–200 MPa pressure range. In agreement with the evidence from Cailliez *et al*.^8^, more hydrophilic zeolites (*i.e*. presence of stronger dipoles on the pore surface) are characterized by infiltration pressures shifted towards lower values (see Fig. 7a). Noteworthy, the infiltration isotherm directly obtained from MD data is best-fitted by *E*_INF_ = 3.47 kJ mol^−1^, which is only 3% lower than the value predicted by Eq. 4. Considering the workstations used to perform the abovementioned simulations (2x Dual Intel® Xeon E5-2620v2), the methodology presented in Fig. 6b has the potential to reduce the computational burden needed to compute one infiltration isotherm by more than one order of magnitude, namely from ~5000 to ~300 CPU hours in this case.

Differently from the first case, where the silanol defects were randomly distributed in the MFI crystal (*i.e*., *ρ*_def_(*z*) = cost, being *ρ*_def_(*z*) the density of defects along the *z* axis), in the second case we analyze a Stripes Distribution of defects also at 0.89% Al/Si (SD, inset of Fig. 7a). Again, the methodology in Fig. 6b allows estimating *E*_INF_ by means of a single MD run. In particular, the computed average water-zeolite energy interaction for *ϑ*_M_ = 0.95 leads to *E*_INF_ = 3.30 kJ mol^−1^ through Eq. 4. The complete set of MD infiltration simulations in Fig. 7a confirms that the SD distribution induces a slight enhancement in the zeolite hydrophilicity, with a 13% increase of *E*_INF_ parameter respect to the random distribution (3.46 vs. 3.05 kJ mol^−1^). The infiltration increase given by SD defect distribution can be due to the localized enhancement of hydrophilicity provided by the defects in the central part of the framework, which promotes the creation of clusters of water molecules easing the water infiltration process (see Fig. 7b). As evident, the prediction capability of Eq. 4 is again demonstrated to be good, being only 5% the discrepancy between the predicted and actual *E*_INF_ value.

## Conclusions

In this article, the infiltration of water into MFI zeolites characterized by different concentration of hydrophilic defects is studied by atomistic simulations validated upon previous experimental results. The introduction of defects in an initially hydrophobic MFI crystal (silicalite-I) allows controlling the hydrophilicity of the zeolite and thus the characteristic water infiltration.

Experimental evidence from the literature showed that the characteristic water infiltration pressure in the MFI zeolites decreases with more hydrophilic pores. The resulting infiltration isotherms can be fitted by a semi-empirical infiltration model similar to the Dubinin-Astakhov’s one for adsorption (see Eq. 1), considering *E*_INF_ and *n*_INF_ as purely tuning parameters. These previous experiments are here employed also to validate an atomistic model for water infiltration in MFI zeolite. Thanks to this validated simulation setup, this work identifies – for the first time – a mechanistic correlation between the chemical characteristics of the zeolite surface (*i.e*. defect concentration, distribution and type) and the *E*_INF_ and *n*_INF_ parameters, which are no more considered as tuning parameters but take a physical-chemical meaning. In detail, *E*_INF_ is demonstrated to have a strong dependence on the interaction energy between zeolite surface and infiltrated water molecules; whereas, *n*_INF_ on the geometrical structure of the zeolite.

This novel mechanistic relationship between the energy of water-zeolite interaction and the parameters of the infiltration model in Eq. 1 is finally employed to explore strategies for regulating the infiltration pressure at a given defects concentration, namely by either introducing more hydrophilic defects or tailoring their local distribution. The suggested methodology is demonstrated to be an accurate tool for reducing more than one order of magnitude the computational time needed to perform extensive sensitivity analyses on geometrical, physical and chemical degrees of freedom of zeolite crystals. The effort, hence, is to provide model-driven guidelines towards the development of advanced materials for zeolite-based devices with the possibility to accumulate, restore and dissipate mechanical energy, as well as for desalination systems based on highly permeable and selective zeolite membranes.

## Methods

### Molecular dynamics geometries

The framework of MFI zeolite is similar to that of both small-pore LTA (Linde Type A) and large-pore FAU (Faujasite) ones, but it has nanopores with intermediate sizes^62^. MFI has an orthorhombic crystal structure (Pnma space group), with *a* = 20.022 Å, *b* = 19.899 Å and *c* = 13.383 Å lattice constants^63^. Zeolites of MFI type have a 45% porosity arising from a 3-dimensional network of channels, which is given by the superimposition of both zig-zag nanopores parallel to [001] direction and straight nanopores parallel to [010] direction. The average diameter of pores is 5.6 Å, whereas channel intersections present cavities with 6.36 Å diameter.

The infiltration isotherms of water in MFI crystals are computed following the numerical protocol previously described by Fasano *et al*.^38^. Here, a membrane made of 2 × 3 × 3 crystal cells of MFI zeolite with dimensions 4 × 6 × 4 nm^3^ is considered, with periodicity along *x* and *y* axis. The pristine MFI crystal without any defects is also known as silicalite-I, and it presents an hydrophobic behavior^50^. Inspired by the “silanol nests model” suggested by Cailliez and colleagues^8,33^, MFI membranes with growing hydrophilicity are here obtained by progressively inserting silanols in the pristine structure, with a random distribution among the possible crystallographic sites. The increased hydrophilicity of the zeolite framework provided by silanols can be related to the concentration of aluminum defects in the MFI structure: the introduction of Al atoms in silicalite-I promotes the presence of dangling oxygens, which in turn form silanol terminals in the structure. Two 4 × 6 × 15 nm^3^ boxes of TIP4P water molecules under ambient conditions (*T* = 300 K, *p* = 1 bar, *ρ* = 1 g cm^−3^, ≅30000 molecules on average) are then put in contact with the *x* − *y* planes of the dry zeolite membrane, thus obtaining the initial computational domain for the infiltration experiments. Note that the most external zeolite surface on the *x* − *y* planes is functionalized by silanols to mimic surface oxidation at the membrane-liquid interface.

Concerning the simulations of zeolite bulk crystals, the computational domain is made of 5 × 5 × 4 silicalite-I unit cells (10.0 × 9.9 × 5.4 nm^3^, periodic boundary conditions along the three Cartesian axis) to guarantee good statistics at low pore hydrations. Starting from the pristine hydrophobic framework, silanols are again progressively inserted to increase the pore hydrophilicity. Finally, zeolite pores are hydrated by a Monte Carlo-like algorithm.

Further details on the simulated geometries can be found elsewhere^38,39^.

### Molecular dynamics force field

Both bonded and nonbonded interactions are modelled in the considered molecular dynamics force field. Bonded interactions take into account the chemical bonds within the zeolite framework, and are mimicked by stretch and angle harmonic potentials^64^. Nonbonded interactions are instead modelled by Coulomb and 12-6 Lennard-Jones potentials for electrostatic and van der Waals interactions, respectively. Particularly, the partial charges of silanols are tuned to fit the infiltration experiments of water in silicalite-I^33,34^, namely *q*_Si_ = 1.4 e, *q*_O_ = −*q*_Si_/2 and *q*_H_ = *q*_Si_/4. TIP4P model^65^ is used for water molecules, as also reported in previous studies about water infiltration in MFI^8^. A twin-range cut-off with shift function is used for the Lennard-Jones potentials; the Particle-Mesh Ewald algorithm with long range dispersion corrections is instead chosen for the Coulomb interactions^66^. Further details, discussions and the complete list of force field parameters are reported in previous works^8,38,39^.

### Molecular dynamics protocol

Both zeolite membranes and bulk crystals are initially energy minimized (steepest descent algorithm). Velocities of atoms are then initialized according to Maxwell distribution (300 K). The energy minimized structure is subsequently hydrated and equilibrated by means of multiple canonical (300 K; Berendsen thermostat) and isothermal-isobaric (300 K, 1 bar; Berendsen thermostat and barostat) ensembles, with up to 1.5 ns trajectories^67^. Zeolite membrane simulations are finally carried out in the isothermal-isobaric ensemble (300 K, infiltration pressure to be tested; velocity rescaling thermostat with 0.1 ps time constant^68^ and isotropic Parrinello-Rahman barostat with 2 ps time constant^69^), with water molecules progressively infiltrating through the initially empty membrane. Note that only the innermost crystal cells of the membrane are accounted for measuring water uptake, to avoid possible artifacts due to the broken crystallinity at the membrane surface^70^. Simulations are continued up to 10–35 ns, when water uptake converges to a steady state value and thus equilibrium conditions are fully achieved. Up to three repetitions are performed per each simulation, and results averaged. Bulk zeolite simulations, instead, are carried out for 2 ns in the canonical ensemble (300 K; velocity rescaling thermostat with 0.1 ps time constant^68^) under equilibrium conditions. Atomistic simulations are performed by GROMACS software (2 fs time step; leap-frog algorithm)^71,72^. Further details on the simulation protocol can be found elsewhere^38,39^.

## Data Availability

The data that support the findings of this study are available from the corresponding author upon request.
